# Efficacy and safety of optic nerve sheath fenestration in patients with raised intracranial pressure

**DOI:** 10.12669/pjms.332.11937

**Published:** 2017

**Authors:** Muhammad Amer Yaqub, Mohammad Asim Mehboob, Qamar Ul Islam

**Affiliations:** 1 Prof. Muhammad Amer Yaqub, MCPS, FCPS, FRCS. Armed Forces Institute of Ophthalmology, Rawalpindi, Pakistan; 2 Dr. Mohammad Asim Mehboob, MBBS. PNS Shifa Naval Hospital, Karachi, Pakistan; 3 Dr. Qamar Ul Islam, MCPS, FCPS(Ophth), FCPS (Vitreoretina). PNS Shifa Naval Hospital, Karachi, Pakistan

**Keywords:** Optic nerve sheath fenestration, Papilledema, Idiopathic Intracranial hypertension

## Abstract

**Objective:**

To evaluate the efficacy and safety of Optic Nerve Sheath Fenestration (ONSF) in patients with raised intracranial pressure (ICP).

**Methods:**

This Quasi Experimental Study was conducted at Armed Forces Institute of Ophthalmology, Rawalpindi from July 2013 to July 2015. Thirty one eyes of 18 patients who underwent ONSF for raised ICP were followed up for one year to ascertain efficacy and safety of ONSF procedure.

**Results:**

Thirteen (72.22%) patients underwent ONSF bilaterally, while five (27.78%) underwent unilateral ONSF. Best corrected visual acuity (BCVA) improved in 24 (77.4%), remained stable in four (12.9%) and deteriorated in three (9.7%) patients. Papilledema improved in 27 (87.1%) while remained stable in four (12.9%) according to Frisénscale of Papilledema. Change in BCVA and papilledema from pre-operative values was statistically significant (p<0.001). There was significant negative correlation (r= -0.434, p=.017) between duration of symptoms before presentation and improvement in BCVA. Common complications were a tonic pupil, subconjunctival haemorrhage, chemosis, weakness of recti and diplopia.

**Conclusion:**

ONSF is an effective procedure with statistically significant improvement in BCVA and reduction in severity of papilledema.

## INTRODUCTION

Raised Intracranial Pressure (ICP) is a cause of significant reduction in vision, and vision related quality of life. Pathogenesis includes increased Cerebro Spinal Fluid (CSF) production, decreased drainage, intracranial space occupying lesions, traumatic cerebral contusion, venous outflow obstruction, dural sinus thrombosis and idiopathic intracranial hypertension (IIH).^1^ IIH is a separate entity, mainly a diagnosis of exclusion, characterized by papilledema without identifiable neurological pathology, and is common in obese women of child bearing age.^2^ Since the optic nerve sheath can be traced as continuation of the subarachnoid space, transmission of effects of raised ICP to the optic nerve causes disruption of the axoplasmic flow, swelling of axons, leakage of water and proteins with resultant optic disc swelling, or papilledema.^3^,^4^ Papilledema, if left untreated, causes significant and irreversible visual loss, with Visual Field (VF) defects and loss of contrast sensitivity.^5^

Treatment of papilledema is primarily focused on treating recognizable causes of raised ICP. IIH is mainly treated by both medical and surgical options. Medical treatment includes Acetazolamide, Steroids, Topiramate, Frousemide and surgical treatment options include Optic Nerve Sheath Fenestration (ONSF) or CSF shunt (lumboperitoneal shunts, ventriculo-peritoneal shunts, or ventriculo-atrial shunt).^3^,^6^,^7^

ONSF, originally described by DeWecker in 1872, is a promising interventional treatment modality which has proved effective in prevention of visual and VF loss.^8^ Hayreh, besides contributing towards description of blood supply of optic nerve, also described the efficacy of ONSF in resolving papilledema.^9^ The literature shows varied efficacy of ONSF procedure. While improvement in vision and papilledema highlights the efficacy, side effects undermine the efficacy of the procedure. ONSF has also been performed in cases of sight threatening papilledema secondary to dural sinus thrombosis or intracranial mass. This study was aimed at evaluating the efficacy and complications of ONSF procedure, in one year follow period in patients with raised ICP.

## METHODS

After approval by the hospital ethical review committee, informed written consent was taken from the patients prior to inclusion in the study. Patients aged between 25-50 years, with Best Corrected Visual Acuity (BCVA) of 6/60 or less on Snellen’s visual acuity chart, progressively decreasing BCVA or VF, non-compliance to medical therapy, refractory headache, established papilledema according to Frisen staging and evidenced on fundoscopy were included.^10^ Patients with pregnancy, deranged renal functions, bleeding disorders, glaucoma, abnormal plasma homocysteine, C-reactive protein and antinuclear antibody levels, use of anticoagulants, infection at surgery site, nystagmus and diagnosed with autoimmune diseases were excluded. All patients were regularly reviewed by Neuro-physician and necessary neuro imaging was done to ascertain the cause of raised ICP like trauma, intracranial mass or dural sinus pathology. Patients continued receiving the medical or surgical therapy advised by neuro-physician and neuro-surgeon. All patients were counselled regarding ONSF surgery and its possible complications.

### Procedure

All patients were operated by technique originally described by Galbraith and Sullivan with slight modification.^11^ Surgery was performed under general anaesthesia. Following medial peritomy, medial rectus was detached from sclera and eyeball was rotated laterally. Optic nerve was approached, and retro-orbital part exposed adequately using either retractor or cotton wool buds on stick. A linear, parallel to optic nerve incision was given to dural sheath and gush of CSF was noted and aspirated. The muscle was re-attached and peritomy closed. Post procedure, all patients received topical Moxifloxacin 0.5%, 6 hourly for 7 days and Prednisolone acetate 1%, 8 hourly for two weeks. The patients were reviewed on first post-operative day, after 7 days, and monthly for 12 months. All procedures were performed by single surgeon to exclude bias.

The principal outcomes included change in BCVA and Frisen Papilledema staging at one year follow up. Efficacy of the procedure was defined as improvement of BCVA from pre-operative level at 12 months follow up visit. All per-operative and post-operative adverse events were recorded to analyse the safety profile of the procedure. The pre devised proforma was completed by single researcher endorsing subject’s demography, ocular examination findings and outcome measures. For statistical analysis, Snellen BCVA was converted to log MAR value using online conversion calculator.

### Statistical Analysis

Statistical package for social sciences (SPSS 17.0) for windows was used for statistical analysis. Descriptive statistics i.e. mean ± standard deviation for quantitative values (age, BCVA, Frisen Staging) and frequencies along with percentages for qualitative variables (gender, laterality of eyes, presenting complaints, etiology of papilledema, complications) were used to describe the data. Shapiro Wilk’s test was used to check normality of data. Post normality testing, paired t’ test was used to compare pre-operative BCVA and Frisen stage from post-operative values. Correlation of nominal variables with improvement in BCVA and Papilledema stage was done using Chi Square test, and correlation of ordinal variables with improvement in BCVA and Papilledema stage was done using one sample t test. P value < 0.05 was considered statistically significant.

## RESULTS

**Fig.1:**
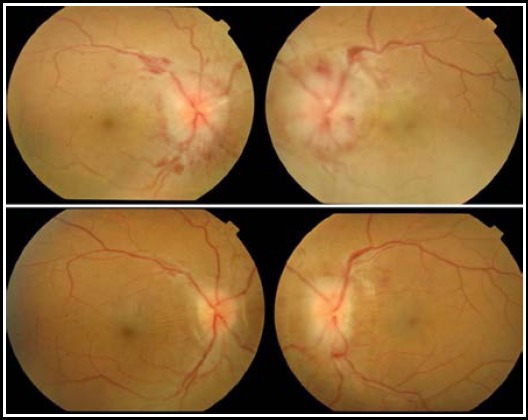
Pre-operative and Post-operative Fundus Photographs of patient who underwent bilateral ONSF

**Fig.2:**
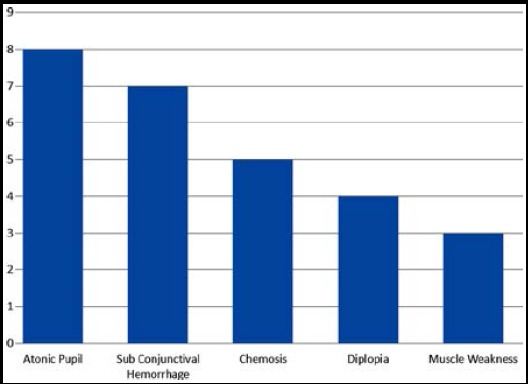
Complications of ONSF (n=31)

Thirty One eyes of 18 patients meeting the inclusion criteria were analysed. Six (33.33%) patients were males, and 12 (66.67%) were females. Thirteen (72.22%) patients underwent ONSF bilaterally, while five (27.78%) underwent unilateral ONSF. Mean age of study population was 36.23 ± 5.19 years (Range 28-46 years). Mean time from on set of symptoms to presentation in ophthalmic Outpatient department was 2.50 ± 1.8 months (Range 1-7 months). Clinical profile of all patients is given in Table-I. BCVA improved in 24 (77.4%), remained stable in 4 (12.9%) and deteriorated in 3 (9.7%) patients. Papilledema improved in 27 (87.1%) while remained stable in four (12.9%) patients according to Frisén Scale of Papilledema. Mean pre-operative and post-operative BCVA and papilledema stage, with mean change is given in Table-II. Change in BCVA and papilledema from pre-operative values was statistically significant (p<0.001). There was significant and negative correlation (r=-0.434, p=.017) between duration of symptoms before presentation and improvement in BCVA. Whereas, correlation of mean change in BCVA and papilledema stage with age, gender, etiology, pre-operative BCVA and pre-operative papilledema stage were not statistically significant. Fig.1 shows pre-operative and post-operative fundus photographs of a patient who underwent bilateral ONSF. Fig.2 shows common complications observed after the procedure.

**Table-I:** Clinical profile of Study population

*Parameter*	*No of patients/ Eyes*
Symptoms (n=18)*	
Headache	15 (83.33%)
Transient Visual obscuration	8 (44.44%)
Nausea/Vomiting	4 (22.22%)
Diplopia	3 (16.67%)
***Etiology (n=18)***	
IIH	11 (61.11%)
Dural Sinus Thrombosis	4 (22.22%)
Intracranial Mass	3 (16.67%)
***BCVA (n=31)***	
6/60	1 (3.20%)
5/60	5 (16.10%)
4/60	5 (16.10%)
3/60	6 (19.50%)
2/60	9 (29.00%)
1/60	5 (16.10%)
***Frisen Stage (n=31)***	
5	3 (9.70%)
4	15 (48.40%)
3	13 (41.90%)

* Occurrence of multiple symptoms in few patients account for percentage distribution of >100%

**Table-II:** Pre and Post-operative Results (n=31)

*Parameter*	*Pre-op Value*	*Post-Op Value (1 year)*	*Mean Change*	*p Value*
BCVA(logMAR)Mean ± SD	1.21 ± 0.19	0.89 ± 0.31	0.31 ± 0.30	< 0.001
Papilledema StagingMean ± SD	3.68 ± 0.65	1.94 ± 1.2	1.74 ± 0.96	< 0.001

## DISCUSSION

Previously, studies on ONSF were mainly focused on treatment of IIH only. Since raised ICP has multiple etiologies, the ONSF has been also been used less frequently for management of other conditions adversely affecting the vision. It has been used in treatment of progressive non-arteritic ischemic optic neuropathy, and optic disc drusen.^12^ It has also been used in case of cryptococcal meningitis secondary to acquired immunodeficiency syndrome successfully.^13^ We used ONSF effectively for treating papilledema secondary to dural sinus thrombosis and intracranial masses, besides IIH, causing sufficient raised ICP to cause visual deterioration. Since ONSF doesn’t affect the integrity of cranial cavity, while only relieving pressure on optic nerve, the procedure can be effectively used in multiple conditions causing raised ICP and papilledema.

Visual acuity is primarily the function of fovea, and does necessarily give insight into preservation of central or paracentral VF.^14^ Still, the efficacy of the procedure has been widely assessed by comparing visual acuity from pre-operative values. Raised ICP significantly affects macula, due to swelling of retinal nerve fibre layer and presence of exudates and subretinal fluid. Resolution of papilledema is therefore expected to improve vision in patients undergoing ONSF. Studies analysing the histological features of IIH have shown that vision loss from outer retinal layer changes in the macula is typically reversible, while vision loss from optic neuropathy and inner retinal layer change is less reversible.^15^ We also didn’t find any significant correlation between mean change in BCVA and stage of papilledema or pre-operative BCVA. Improvement in BCVA in our patients did not depend upon pre-operative papilledema stage, explaining the independence of macular function from optic disc swelling alone. Also, mean change in BCVA was independent from pre-operative BCVA, explaining that poor vision before surgery doesn’t necessarily demerit the performance of surgery.

The improvement in BCVA after ONSF is a debatable subject. Studies have shown wide range in improvement in BCVA, from as low as 14% to as high as 100%.^17^,^18^ Our study showed improvement in BCVA in 24 (77.4%), stability in 4 (12.9%) and deterioration in 3 (9.7%) eyes. In the largest study conducted on 578 eyes of 331 patients, improvement or stability was seen in 94.4% and worsening in 5.6% of eyes.^12^

ONSF is primarily meant to improve vision only, but studies have shown efficacy of ONSF in improvement of symptoms also. This is evidenced by improvement in headache in patients undergoing the procedure. Literature shows wide range in improvement of headache, with as low as 13%, and as high as 90% patients showing improvement in headache.^19^,^20^ Twelve of our patients (66.67%) showed improvement in headache. Out of these 12 patients, 10 had undergone ONSF bilaterally. So there is indirect evidence from improvement in headache in 10 out of 13 patients undergoing ONSF bilaterally, that ONSF is also helpful in controlling chief symptom of IIH or raised ICP.

We observed papilledema improvement in 27 (87.1%) eyes, and stable papilledema staging in four (12.9%) eyes. Results are comparable to studies done earlier, with improvement of papilledema in range of 71 to 100%.^21^,^22^ It is pertinent to mention here that improvement in papilledema stage does not necessarily mean complete resolution of edema. None of the eyes showed complete resolution of edema, while 15 eyes (48.4%) showed Frisen Stage 1 papilledema after surgery. In the largest meta-analysis done on efficacy of ONSF, including 8 studies, and 432 cases with follow up of 20 months, mean frequency of improvement in headache, visual acuity and papilledema was 26%, 42% and 92% respectively.^3^ Our results are comparable to the analysis, with significantly more cases showing improvement in headache (66.67%) and BCVA (77.4%).

Due to the delicate nature of surgery, safety remains one of the major concerns of the procedure. The safety profile of ONSF procedure was satisfactory. Our study showed atonic pupil, subconjunctival hemorrhage, chemosis, diplopia and muscle weakness as the major complications of the procedure. Most of the complications were transient in nature, with only two eyes showing atonic pupil and one patient reporting diplopia in primary gaze after 12 months follow up period. The ONSF is a relatively safe procedure with agreement in complication rate ranging from 5 to 45% in literature.^23^ Banta JT et al reported severe, vision-limiting surgical complication in only one eye (<1%).^19^ Corbett JJ et al showed 16 eyes with atonic pupil after ONSF, which remained permanent. Complications like retrobulbar hemorrhage and sixth nerve palsy have been reported in the literature.^24^ Other less frequently observed complications were transient blindness, choroidal infarction, diplopia and orbital infections.^12^,^25^ Shorter follow up time and no comparison of VF defects between pre and post-operative eyes are few limitations of the study. Longer follow up is required to ascertain long term efficacy and safety of ONSF procedure.

## CONCLUSION

ONSF is a safe and effective procedure for managing severe visual deterioration in cases of raised ICP due to IIH, dural sinus thrombosis and intracranial mass.

### Authors’ Contribution

**MAY** conceived, designed, performed surgery, reviewed and finally approved manuscript.

**MAM** did data collection, statistical analysis, manuscript writing.

**QUI** did statistical analysis and manuscript editing.
